# Secular trends in physical fitness of rural Chinese children and adolescents aged 7–18 years from 1985 to 2019

**DOI:** 10.1038/s41598-023-31190-x

**Published:** 2023-03-14

**Authors:** Chengyue Li, Alimujiang Yimiti Taerken, Qian Li, Adilijiang Selimu, Hao Wang

**Affiliations:** Institute of Physical Education, Xinjiang Normal University, Xinjiang Uygur Autonomous Region, Urumqi, 830054 China

**Keywords:** Health care, Health occupations

## Abstract

The main purpose of the study was to evaluate the secular trends in physical fitness of Chinese rural children and adolescents aged 7–18 from 1985 to 2019. The speed, muscular strength, explosive power fitness, cardiorespiratory fitness, and flexibility were investigated by National Survey on Students’ Constitution and Health in 1985, 2000, 2010 and 2019. During the period 1985–2000, the physical fitness of Chinese rural children and adolescents improved except for flexibility, and almost all of them reached the maximum increase rate. During the period 2000–2010, in addition to the improvement of flexibility, other fitness showed downward trends, and the decline ranges were large. During the period 2010–2019, the speed of boys rebounded, flexibility, explosive power and muscular strength continued to decrease. Meanwhile, speed, flexibility and muscular strength in girls rebounded, and the explosive power continued to decline. From 2000 to 2019, the body mass index increase accelerated. This study shows that some components of physical fitness of Chinese rural children and adolescents have shown positive trends in recent years, especially for girls and adolescents aged 13–15 years. However, it may also imply inequality between sexes and ages, which provides a reference for the focus of the country's physical fitness and health monitoring and intervention measures.

## Introduction

Physical fitness is a multicomponent construct that is closely related to the ability to perform physical activity^1^. Although its measurement varies from country to country, core items usually include endurance running (reflecting cardiorespiratory fitness), standing long jump (reflecting explosive power), 1-min pull-ups and sit-ups (reflecting muscular strength), sit-and-reach (reflecting flexibility), and 50-m (or 60-m) dash (reflecting speed)^2^. These tests cover different functions and structures of the body`s movement, including musculoskeletal, cardiorespiratory, circulatory, endocrine metabolic and psycho-neurological functions^2^. Given the current concerns about the declining fitness of the world's children and adolescents and its potential association with nutritional and health status in adulthood^3,4^, cardiovascular fitness is often the focus of attention, with its association with greater BMI and fat mass^5^, while speed, flexibility and strength performances are core fitness for children and adolescents to participate in multiple forms of physical activity^6^.

In China, the "Research on Physical Shape, Function and Fitness of Chinese Children and Adolescents" project started in 1979^7^, which was the first national growth and development survey performed by the former state Physical Culture and Sports Commission, covering only 16 provinces/municipalities directly under the central government^8^. Starting in 1985, five central ministries and commissions, including the Ministry of Education, established the "National Students fitness and Health Survey" system, in which almost all provinces, autonomous regions, and municipalities directly under the central government participated in the study once every five years and continued to increase the scope of the study afterward. Since 1985, eight surveys have been performed, providing comprehensive scientific data on the physical development of children and adolescents in China, and many achievements have been made during this period (the latest survey was conducted in 2019). Since the “reform and opening of China” in 1978, the national economy has increased rapidly, and the primary manifestation of this was the improvement of people's material life. The level of diet and nutrition of children and adolescents has improved significantly, the growth potential has been stimulated, and physical fitness has begun to improve. However, since around the twenty-first century, the height development of Chinese children and adolescents has slowed down while their weight has increased dramatically^9^, and the situation of overweight and obesity has become serious^10^, and physical fitness has begun to decline or fluctuate^2,11^.

Previous studies have shown that only speed and flexibility rebounded among Chinese children and adolescents in 2014, while other fitness components continued to decline to vary degrees, and although the urban–rural gap decreased, the advantage of rural children and adolescents in endurance fitness and explosive strength also narrowed^11,12^. Meanwhile, recent regional studies have shown that the physical fitness of children and adolescents in rural areas, although better than in urban areas, has been on a decreasing trend^13^. However, these findings do not reflect the Chinese overall situation. Current national studies of secular trends in children and adolescents’ physical fitness are mainly time-scale changes, rely on cross-sectional designs, usually compare only a few time points for children and adolescent age groups or joint age groups and have small sample sizes^12,14–16^. Recently, several systematic reviews on the secular trend of cardiorespiratory fitness and muscular strength of children and adolescents have indicated that in recent years, muscular strength, measured by grip strength, has improved in some countries^17^, the changes in cardiorespiratory fitness, measured by 20-m shuttle run, have tended to be stable^18^, and muscular endurance, measured by sit-ups^19^ and explosive power, measured by standing long jump, have worsened^20^. Although these analyses provide the secular trends of children and adolescents in China, the report does not distinguish the differences between urban and rural areas, lacks some test items, and the data are relatively old. In recent years, more attention has been given to research on muscular strength and cardiorespiratory fitness^16–21^, although due to its significant role in health and recent negative trends worldwide^6,15,18–21^, there is still a lack of comprehensive research on some health-related physical fitness, especially speed and flexibility fitness.

An inverted U-shaped relationship between body mass index (BMI) and physical fitness has been shown, suggesting that malnutrition and overweight obesity could have a negative impact on physical fitness^2,22,23^. In 1985, the prevalence of malnutrition among rural children and adolescents was as high as 24.2%, approximately 2.7 times higher than that in urban areas, but the prevalence of overweight and obesity was less than 1.0%^24^. Subsequently, China actively carried out programs for "rural revitalization"^25^, physical education reform, and the “nutrition improvement program for rural students in compulsory education”^26^ and so on. Malnutrition in rural areas has been greatly improved, and physical fitness have improved, but recent studies have shown that obesity among rural children and adolescents has increased dramatically, the increasing rate of obesity in rural areas is greater than that in urban areas^27^, and too many obese individuals in rural areas can also lead to a decline in physical fitness. In addition, the increase in sedentary time and the decrease in physical activity of children and adolescents in recent years has also led to a negative impact on physical fitness^28^. In summary, it is necessary to carry out secular trends research on rural children and adolescents in China, which can not only provide a reference for Chinese physical health, physical education and public health policies but also make efforts to supplement the research on physical fitness in recent years and guide future global physical health research and health monitoring^29^.

Therefore, this paper conducts an analysis of the secular trends of Chinese children and adolescents’ physical fitness in rural areas over a 34-year period by the Chinese National Surveillance on Students' Constitution and Health (CNSSCH). Specifically, we aim to (1) investigate the secular trends of physical fitness of five fitness components of Chinese children and adolescents aged 7–18 years in rural during the entire period, (2) understand the changes between subgroups (age, sex) and different periods, so as to find inequalities in the health of Chinese children and adolescents.

## Methods

### Study design and subjects

Data were obtained from test scores of Han Chinese children and adolescents aged 7–18 in rural areas by CNSSCH^30–33^ in 1985, 2000, 2010, and 2019. CNSSCH was organized by the Ministries of Education, Health, Science and Technology, the State Ethnic Affairs Commission, and the State Sports General Administration of the People’s Republic of China^30–33^. Multistage stratified cluster sampling was used to maintain consistent sampling and assessment methods across survey years with the class as the sampling unit. The sampling procedure was performed as previously described in detail^2,24^. 29 provinces/autonomous regions/municipalities directly under the central government (34 overall), excluding Hong Kong, Macau, Taiwan, Hainan, and Chongqing, were included in 1985, and Hainan and Chongqing were included in the latter three surveys. This study only included participants of the Han ethnicity, who account for 92% of the total Chinese population, from 26 mainland provinces and 4 municipalities of mainland China, excluding Tibet (where the Han ethnicity is a minority). Since 1985, children and adolescents in each province, except Tibet, were stratified into three levels according to their socioeconomic status (upper, moderate, and low) and then, in turn, stratified by urban and rural areas according to their place of residence, with at least 50 Han Chinese students in each age group included in the survey. The classifications of urban and rural were based on the revised criteria for designated towns issued in 1984^34^. It has not changed since the initial classification in 1985, which means that if an area initially classified as rural experienced urbanization, it remained classified as rural. The exclusion criteria for participants were: (1) suffering from important organ diseases such as heart, lung, liver, and kidney; (2) abnormal physical development (e.g., pygmyism, gigantism); (3) those with physical disabilities or deformities; (4) those with acute illnesses, or those who suffered from acute illnesses in the last month of the testing period and had not recovered their physical strength; (5) girls who were menstruating (The girls were asked about their menstrual status in each age group by the female internist, and only they were asked "with or without" being menstruating). All participants were grouped by sex and age, with 1 year being an age group and 24 age groups in total. Participants with missing data or illogical test results were excluded. From 1985 to 2019, 160,588, 263,421, 262,765, 262,661, 259,757 and 260,448 boys and 160,888, 262,667, 262,847, 262,687, 262,727 and 260,839 girls aged 7–18 were tested for BMI (1985 data missing), speed, explosive power, flexibility, muscular strength and cardiorespiratory fitness, respectively. The number of boys and girls tested in 2000, 2010, and 2019 ranged from 51,000 to 54,000 with a rate of approximately 1:1 for each age group, and the number of those tested in 1985 was approximately twice the other survey years. There were similar numbers in each age group. See Tables 1 and 2 for details.

**Table 1:** Comparison of physical fitness scores of five fitness components of Chinese boys of different age categories from 1985 to 2019.

Age categories(year)	1985 (a)				2000 (b)				2010 (c)				2019 (d)				*F*	Significant post hoc comparisons ^#^	*R*^2^	*B* ^&^
	Age	N	M	SD	Age	N	M	SD	Age	N	M	SD	Age	N	M	SD				
7–12																				
Body mass index (kg/m^2^)					9.49 (1.71)	27,120	15.91	2.33	9.50 (1.71)	26,938	16.97	2.95	9.48 (1.71)	26,833	18.03	3.59	3370.270***	b vs c, b vs d, c vs d	0.999	0.111*
50-m dash (s)	9.50 (1.71)	51,342	10.19	1.09	9.49 (1.71)	27,117	9.97	1.14	9.50 (1.71)	26,896	10.03	1.20	9.48 (1.71)	26,496	10.03	1.28	266.836***	a vs b, a vs c, a vs d, b vs c, b vs d	0.590	− 0.005
Standing long jump (cm)	9.50 (1.71)	51,342	140.84	21.70	9.49 (1.71)	27,156	152.22	24.23	9.50 (1.71)	26,916	149.70	24.75	9.48 (1.71)	26,522	143.58	26.77	1694.614***	a vs b, a vs c, a vs d, b vs c, b vs d, c vs d	0.203	0.158
Stand/sit-and-reach (cm)	9.50 (1.71)	51,342	5.52	4.50	9.48 (1.71)	27,130	4.85	4.80	9.50 (1.71)	26,921	6.44	5.34	9.48 (1.71)	26,730	5.94	6.21	474.044***	a vs b, a vs c, a vs d, b vs c, b vs d, c vs d	0.238	0.019
oblique body pull-ups (n)	9.50 (1.71)	51,342	18.08	10.80	9.47 (1.71)	25,293	29.00	14.78	9.50 (1.71)	26,865	28.57	19.31	9.40 (1.67)	25,597	24.61	20.17	4028.403***	a vs b, a vs c, a vs d, b vs c, b vs d, c vs d	0.526	0.259
50-m × 8 shuttle run (s)	9.50 (1.71)	51,342	114.67	11.58	9.48 (1.71)	27,101	119.56	15.39	9.49 (1.70)	26,795	123.84	17.40	9.41 (1.67)	25,414	126.46	19.25	41,119.917***	a vs b, a vs c, a vs d, b vs c, b vs d, c vs d	0.997	0.353**
13–15																				
Body mass index (kg/m^2^)					14.01 (0.82)	13,290	18.20	2.64	14.00 (0.82)	13,477	19.08	3.01	14.01 (0.82)	13,197	20.41	3.91	1572.592***	b vs c, b vs d, c vs d	0.978	0.116
50-m dash (s)	14.00 (0.82)	25,675	8.65	0.71	14.01 (0.82)	13,277	8.33	0.79	14.00 (0.82)	13,464	8.33	0.92	14.01 (0.82)	13,033	8.09	0.96	1458.596***	a vs b, a vs c, a vs d, b vs d, c vs d	0.943	− 0.015*
Standing long jump (cm)	14.00 (0.82)	25,675	185.07	22.92	14.01 (0.82)	13,305	201.53	24.15	14.00 (0.82)	13,466	199.73	26.28	14.01 (0.82)	13,062	198.30	28.57	1846.750***	a vs b, a vs c, a vs d, b vs c, b vs d, c vs d	0.675	0.446
Stand/sit-and-reach (cm)	14.00 (0.82)	25,675	8.80	5.62	14.01 (0.82)	13,141	7.70	6.05	14.00 (0.82)	13,467	8.89	6.71	14.01 (0.82)	13,125	8.17	7.57	116.352***	a vs b, a vs d, b vs c, b vs d, c vs d	0.126	− 0.012
Pull-ups (n)	14.00 (0.82)	25,675	3.23	3.17	14.01 (0.82)	13,294	4.37	4.50	14.00 (0.82)	13,441	3.45	4.85	14.01 (0.82)	12,977	2.94	4.48	313.363***	a vs b, a vs c, a vs d, b vs c, b vs d, c vs d	0.009	− 0.003
1000-m run (s)	14.00 (0.82)	25,675	255.35	24.57	14.01 (0.82)	12,915	268.28	33.11	14.01 (0.82)	13,216	284.21	40.81	14.01 (0.82)	12,962	277.26	47.47	2320.016***	a vs b, a vs c, a vs d, b vs c, b vs d, c vs d	0.844	0.789
16–18																				
Body mass index (kg/m^2^)					17.01 (0.82)	13,414	19.88	2.44	17.00 (0.82)	13,442	20.25	2.77	16.98 (0.82)	12,877	21.58	3.77	1135.429***	b vs c, b vs d, c vs d	0.882	0.088
50-m dash (s)	17.00 (0.82)	25,640	7.88	0.56	17.01 (0.82)	13,398	7.58	0.58	17.00 (0.82)	13,401	7.68	0.74	16.98 (0.82)	12,782	7.67	0.87	702.666***	a vs b, a vs c, a vs d, b vs c, b vs d	0.556	− 0.007
Standing long jump (cm)	17.00 (0.82)	25,640	212.69	20.12	17.01 (0.82)	13,423	227.59	19.96	17.00 (0.82)	13,435	226.26	22.19	16.98 (0.82)	12,823	220.00	25.60	1890.707***	a vs b, a vs c, a vs d, b vs c, b vs d, c vs d	0.426	0.311
Stand/sit-and-reach (cm)	17.00 (0.82)	25,640	13.20	5.84	17.01 (0.82)	13,239	11.35	6.90	17.00 (0.82)	13,431	12.55	6.96	16.98 (0.82)	12,820	10.91	7.64	434.280***	a vs b, a vs c, a vs d, b vs c, b vs d, c vs d	0.623	− 0.056
Pull-ups (n)	17.00 (0.82)	25,640	6.90	3.88	17.01 (0.82)	13,417	7.34	4.52	17.00 (0.82)	13,392	5.49	5.31	16.98 (0.82)	12,814	4.33	4.74	1338.465***	a vs b, a vs c, a vs d, b vs c, b vs d, c vs d	0.702	− 0.07
1000-m run (s)	17.00 (0.82)	25,640	233.04	20.42	17.01 (0.82)	13,251	243.80	24.47	17.00 (0.82)	13,417	255.84	31.56	16.98 (0.82)	12,720	262.83	39.26	3894.106***	a vs b, a vs c, a vs d, b vs c, b vs d, c vs d	0.991	0.886**
All																				
Body mass index (kg/m^2^)					12.48 (3.46)	53,824	17.46	2.96	12.50 (3.45)	53,857	18.31	3.24	12.44 (3.45)	52,907	19.49	4.02	4703.305***	b vs c, b vs d, c vs d	0.985	0.106
50-m dash (s)	12.50 (3.45)	102,657	9.23	1.34	12.48 (3.46)	53,792	8.97	1.41	12.50 (3.45)	53,761	9.02	1.46	12.44 (3.45)	53,211	8.97	1.56	622.830***	a vs b, a vs c, a vs d, b vs c, c vs d	0.774	− 0.008
Standing long jump (cm)	12.50 (3.45)	102,657	169.85	37.49	12.48 (3.46)	53,884	183.17	39.96	12.50 (3.45)	53,817	181.33	41.12	12.45 (3.45)	52,407	175.92	43.08	1710.962***	a vs b, a vs c, a vs d, b vs c, b vs d, c vs d	0.388	0.259
Stand/sit-and-reach (cm)	12.50 (3.45)	102,657	8.26	6.04	12.46 (3.46)	53,510	7.16	6.29	12.50 (3.45)	53,819	8.58	6.62	12.44 (3.45)	52,675	7.71	7.22	536.586***	a vs b, a vs c, a vs d, b vs c, b vs d, c vs d	0.045	− 0.008

N, is the sample size; M, is the mean, and SD is the standard deviation.

#One-way analysis of variance (ANOVA) with the Bonferroni post hoc test.

&Sample-weighted linear regression.

*Represents *p* < 0.05; **represents *p* < 0.01; ***represents *p* < 0.001, same below.

**Table 2:** Comparison of physical fitness scores of five fitness components of Chinese girls of different age categories from 1985 to 2019.

Age categories(year)	1985 (a)				2000 (b)				2010 (c)				2019 (d)				*F*	Significant post hoc comparisons	*R*^2^	*B*
	Age	N	M	SD	Age	N	M	SD	Age	N	M	SD	Age	N	M	SD				
7–12																				
Body mass index (kg/m^2^)					9.50 (1.71)	26,990	15.63	2.35	9.50 (1.71)	26,971	16.35	2.56	9.51 (1.71)	26,969	17.30	3.13	2596.387***	b vs c, b vs d, c vs d	0.988	0.088
50-m dash (s)	9.50 (1.71)	51,341	10.71	1.20	9.50 (1.71)	26,964	10.53	1.23	9.50 (1.71)	26,952	10.61	1.20	9.50 (1.71)	26,642	10.46	1.21	291.723***	a vs b, a vs c, a vs d, b vs c, b vs d, c vs d	0.767	− 0.006
Standing long jump (cm)	9.50 (1.71)	51,341	133.08	20.2	9.50 (1.71)	26,995	140.47	22.63	9.50 (1.71)	26,953	137.21	22.77	9.50 (1.71)	26,669	132.77	23.55	860.430***	a vs b, a vs c, b vs c, b vs d, c vs d	0.030	0.039
Stand/sit-and-reach (cm)	9.50 (1.71)	51,341	7.66	4.63	9.50 (1.71)	26,999	6.27	4.86	9.50 (1.71)	26,954	9.46	5.33	9.50 (1.71)	26,710	10.57	6.32	3771.308***	a vs b, a vs c, a vs d, b vs c, b vs d, c vs d	0.524	0.082
1-min sit-ups (n)	9.50 (1.71)	51,341	16.60	10.75	9.50 (1.71)	26,979	24.05	11.30	9.50 (1.71)	26,933	20.67	10.32	9.49 (1.71)	26,595	25.35	10.86	4973.544***	a vs b, a vs c, a vs d, b vs c, b vs d, c vs d	0.728	0.231
50-m × 8 shuttle run (s)	9.50 (1.71)	51,341	121.24	12.29	9.50 (1.71)	26,967	125.73	16.17	9.49 (1.70)	26,770	128.45	16.16	9.43 (1.68)	25,538	130.20	18.10	2495.489***	a vs b, a vs c, a vs d, b vs c, b vs d, c vs d	0.995	0.271**
13–15																				
Body mass index (kg/m^2^)					14.00 (0.82)	13,425	18.61	2.47	14.00 (0.82)	13,458	19.24	2.68	14.00 (0.82)	13,214	20.48	3.33	1484.940***	b vs c, b vs d, c vs d	0.953	0.098
50-m dash (s)	14.00 (0.82)	25,671	9.58	0.81	14.00 (0.82)	13,419	9.50	0.84	14.00 (0.82)	13,422	9.74	1.00	14.00 (0.82)	13,084	9.51	1.03	201.148***	a vs b, a vs c, a vs d, b vs c, c vs d	0.003	0.000
Standing long jump (cm)	14.00 (0.82)	25,671	158.37	17.93	14.00 (0.82)	13,431	166.40	18.62	14.00 (0.82)	13,426	161.13	19.13	14.00 (0.82)	13,100	156.83	21.93	691.314***	a vs b, a vs c, a vs d, b vs c, b vs d, c vs d	0.000	− 0.001
Stand/sit-and-reach (cm)	14.00 (0.82)	25,671	10.34	5.53	14.00 (0.82)	13,276	8.70	5.89	14.00 (0.82)	13,416	11.44	6.39	14.00 (0.82)	13,166	12.71	7.25	1027.921***	a vs b, a vs c, a vs d, b vs c, b vs d, c vs d	0.395	0.062
1-min sit-ups (n)	14.00 (0.82)	25,671	20.97	9.99	14.00 (0.82)	13,431	29.73	10.60	14.00 (0.82)	13,413	25.75	10.12	14.00 (0.82)	13,108	30.60	10.84	3467.113***	a vs b, a vs c, a vs d, b vs c, b vs d, c vs d	0.708	0.257
800-m run (s)	14.00 (0.82)	25,671	231.27	24.12	14.00 (0.82)	13,269	246.96	30.10	14.01 (0.82)	13,418	262.11	33.81	14.00 (0.82)	12,951	256.53	38.70	3704.561***	a vs b, a vs c, a vs d, b vs c, b vs d, c vs d	0.878	0.884
16–18																				
Body mass index (kg/m^2^)					17.00 (0.82)	13,485	20.28	2.31	17.00 (0.82)	13,459	20.29	2.38	16.98 (0.81)	12,917	21.09	3.12	411.715***	b vs d, c vs d	0.727	0.042
50-m dash (s)	17.00 (0.82)	25,550	9.44	0.81	17.00 (0.82)	13,471	9.33	0.86	17.00 (0.82)	13,419	9.69	1.03	16.98 (0.81)	12,732	9.66	1.18	474.788***	a vs b, a vs c, a vs d, b vs c, b vs d	0.508	0.007
Standing long jump (cm)	17.00 (0.82)	25,550	162.53	17.88	17.00 (0.82)	13,497	171.69	17.89	17.00 (0.82)	13,425	166.88	18.72	16.98 (0.81)	12,789	162.70	21.16	822.869***	a vs b, a vs c, b vs c, b vs d, c vs d	0.041	0.055
Stand/sit-and-reach (cm)	17.00 (0.82)	25,550	12.77	5.57	17.00 (0.82)	13,298	10.82	6.26	17.00 (0.82)	13,424	13.58	6.49	16.98 (0.81)	12,882	14.14	7.08	717.771***	a vs b, a vs c, a vs d, b vs c, b vs d, c vs d	0.201	0.038
1-min sit-ups (n)	17.00 (0.82)	25,550	21.17	10.18	17.00 (0.82)	13,493	32.73	9.65	17.00 (0.82)	13,420	27.67	9.91	16.98 (0.81)	12,793	31.68	10.73	5190.252***	a vs b, a vs c, a vs d, b vs c, b vs d, c vs d	0.641	0.296
800-m run (s)	17.00 (0.82)	25,550	229.01	23.00	17.00 (0.82)	13,333	241.89	26.01	17.00 (0.82)	13,437	252.32	28.47	16.99 (0.81)	12,594	257.26	34.12	3903.492***	a vs b, a vs c, a vs d, b vs c, b vs d, c vs d	0.994	0.861**
All																				
Body mass index (kg/m^2^)					12.50 (3.45)	53,900	17.53	3.102	12.50 (3.45)	53,888	18.06	3.0883	12.44 (3.44)	53,100	19.01	3.63	2790.353***	b vs c, b vs d, c vs d	0.963	0.077
50-m dash (s)	12.49 (3.45)	102,562	10.11	1.19	12.50 (3.45)	53,854	9.97	1.20	12.49 (3.45)	53,793	10.16	1.20	12.44 (3.45)	52,458	10.03	1.24	279.091***	a vs b, a vs c, a vs d, b vs c, b vs d, c vs d	0.049	− 0.001
Standing long jump (cm)	12.49 (3.45)	102,562	146.74	23.53	12.50 (3.45)	53,923	154.74	25.11	12.50 (3.45)	53,804	150.58	24.94	12.44 (3.44)	52,558	146.05	26.38	1575.503***	a vs b, a vs c, a vs d, b vs c, b vs d, c vs d	0.014	0.029
Stand/sit-and-reach (cm)	12.49 (3.45)	102,562	9.61	5.53	12.50 (3.45)	53,573	8.00	5.82	12.50 (3.45)	53,794	10.98	6.15	12.45 (3.43)	52,758	11.98	6.99	4468.133***	a vs b, a vs c, a vs d, b vs c, b vs d, c vs d	0.418	0.066
1-min sit-ups (n)	12.49 (3.45)	102,562	18.83	10.66	12.48 (3.45)	53,903	27.64	11.37	12.49 (3.45)	53,766	23.68	10.63	12.45 (3.44)	52,496	28.20	11.21	12,094.560***	a vs b, a vs c, a vs d, b vs c, b vs d, c vs d	0.699	0.252

### Measurements

The height was measured to the nearest 0.1 cm by mechanical height and sitting height meter. The subjects did not wear shoes, and their heel, sacrum, and two shoulder blades were in contact with the column in a "three points and one line" standing posture. The weight was measured to the nearest 0.1 kg by an electronic weight meter or lever scale. The subjects stood barefoot in the center of the weight meter for 3 to 5 s, and the value was recorded. Boys wore shorts and girls wore shorts and short-sleeved shirts. BMI was calculated as weight in kilograms divided by height in meters squared [weight(kg)/height(m)^2^]. Survey participants were given complete physical fitness tests at all survey sites following the same protocol. All physical fitness tests were administered in physical education classes by specially trained physical education teachers who had passed a measurement test. A school physician was present to prevent injuries to children and adolescents during the physical fitness tests, and a program director was present to monitor that the physical fitness tests were conducted as required and to provide the necessary guidance. A group of trained field investigators measured five physical fitness: explosive power (standing long jump, SLJ), speed (50-m dash, D50), flexibility (sit/stand-and-reach, SR), muscular strength, and cardiorespiratory fitness following standardized procedures. The specific test procedures and details are described in previous studies^2,35^. According to the differences in physical fitness by age and sex, muscular strength was assessed by oblique body pull-ups (OPU) for boys aged 7–12, pull-ups (PU) for boys aged 13–18, and 1-min sit-ups (SU) for girls aged 7–18 years. Cardiorespiratory fitness was assessed by 50-m × 8 shuttle run (50SR) for boys and girls aged 7–12, a 1000-m run (1000R) for boys aged 13–18, and an 800-m run (800R) for girls aged 13–18. From 1985 to 2000, the flexibility test was stand-and-reach. For safety reasons, since 2005, sit-and-reach has been used to measure flexibility. All the measuring instruments were consistent in each survey year and calibrated before use. All the students in the final analysis took each test simultaneously. Nearly 100% of participants performed all tests on the same day. Note that smaller values for speed and endurance fitness tests represent better performance, while larger values for other fitness tests represent better performance.

### Statistical analysis

All results for physical fitness were summarized as the mean (M) and standard deviation (SD). Akima splines were used to establish the change curve of each sex-age group, with the x-axis as the years and the y-axis as the results. The sample-weighted linear regression was used to assess the secular trends in means of five fitness components and BMI, with the independent variable being the year and the dependent variable being the test score. The fitting degree was expressed as *R*-squared, and the regression coefficient (*B*) represented the value of annual change. Mean differences among all subgroups were tested by one-way analysis of variance (ANOVA) with the Bonferroni post hoc test to verify significance between every two survey years. The level of statistical significance was set at 0.05. All physical fitness indicators from 1985 to 2019 were divided into 3 stages: 1985–2000 as the 1st stage, 2000–2010 as the 2nd stage, and 2010–2019 as the 3rd stage, and the increased range per decade in each sex-age category were calculated for the 3 stages. The calculation formula is:

$$\begin{aligned} & {\text{Average change value of an indicator per decade }}\left( {\text{/10a}} \right) \\ & \;\;\; = \left( {{\text{average value of the subsequent survey year}} - {\text{average value of the previous survey year}}} \right) \\ & \;\;\;\;\;\;\;/\left( {{\text{subsequent survey year}} - {\text{previous survey year}}} \right) \times 10. \\ \end{aligned}$$

To understand the differences between subgroups (age, sex), data from each survey were divided into 3 age categories, 7–12 years old (primary school), 13–15 years old (junior middle school) and 16–18 years old (junior high school) according to the Chinese educational phases in each sex. Data are processed as above. All analyses were conducted using IBM SPSS version 27.0 (IBM Corp, Armonk, NY, USA) and GraphPad Prism 9.3.1 (GraphPad Software, Inc, CA, USA).

### Ethics statement

The studies involving human participants were reviewed and approved by the Medical Research Ethics Committee of the Peking University Health Science Center (IRB00001052-19095). All participants and guardians participated voluntarily and written informed consent by the participant’ legal guardian/next of kin were obtained before the survey. All methods were performed in accordance with relevant guidelines and regulations (detailed rules and regulations of Chinese National Surveillance on Students' Constitution and Health).

## Results

### BMI

**Figure 1:**
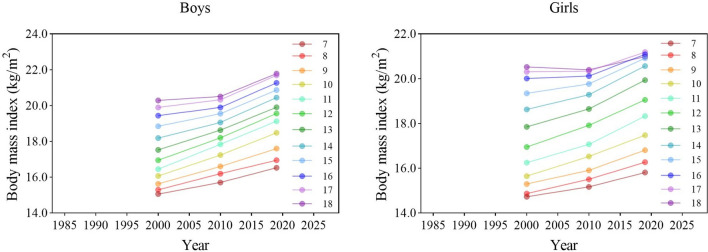
Secular trends (means) in body mass index for Chinese boys and girls aged 7–18 in rural from 1985 to 2019.

During the period 2000–2019, the BMI of boys and girls in all age groups increased (Fig. 1). There were significant differences in the BMI of boys and girls between the three survey years [boys: *F* = 3370.270 (7–12 years), 1572.592 (13–15 years), 1135.429 (16–18 years), 4703.305 (7–18 years); girls: 2596.387 (7–12 years), 1484.940 (13–15 years), 411.715 (16–18 years), 2790.353 (7–18 years), all *p* < 0.001] (Tables 1, 2). In 2019, compared with 2000, the BMI of boys and girls in all age categories increased (all *p* < 0.05). During the period 2000–2010 and 2010–2019, the BMI of boys and girls in general increased (all *p* < 0.05) but there was an exception in that the BMI of girls aged 16–18 years had no significant change (*p* > 0.05) (Tables 1, 2). In terms of the rate of change, the increase accelerated in the 3rd stage for boys and girls in all age categories compared with the 2^nd^ stage, especially in the age of 16–18 (Fig. 7).

### Speed

**Figure 2:**
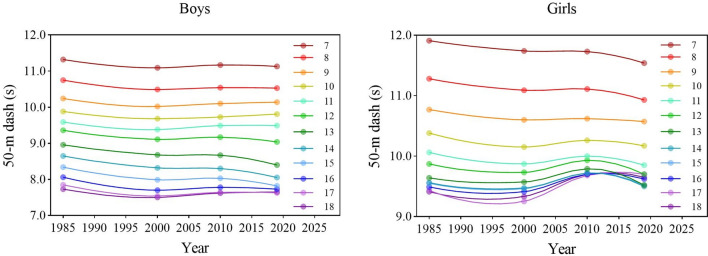
Secular trends (means) in 50-m dash tests for Chinese boys and girls aged 7–18 in rural from 1985 to 2019. Dots and solid lines are the means and Akima splines, respectively. Upward sloping lines represent poorer performance over time and downward sloping lines represent better performance.

During the entire period, the speed of boys and girls in all age groups improved first, then worsened, and finally rebounded slightly, with slight differences among different age groups (Fig. 2). There were significant differences in the results of D50 of boys and girls between the four survey years [boys: *F* = 266.836 (7–12 years), 1458.596 (13–15 years), 702.666 (16–18 years), 622.830 (7–18 years); girls: 291.723 (7–12 years), 201.148 (13–15 years), 474.788 (16–18 years), 279.091 (7–18 years), all *p* < 0.001] (Tables 1, 2). Boys aged 13–15 showed a linear decline in the results of D50 over 34 years (*p* < 0.05), which represented the improvement in speed. During the entire 34-year period, in other words, 2019 compared with 1985, boys' and girls' speed improved (decreased in the results of D50) (all *p* < 0.05) but there was an exception in that the speed of girls aged 16–18 years worsened (*p* < 0.05). From each period, during the period 1985–2000, the speed improved in all age categories and overall for both sexes (all *p* < 0.05); during the period 2000–2010, only speed for boys aged 13–15 years had no significant change (*p* > 0.05), while that others of age categories improved (all *p* < 0.05); during the period 2010–2019, speed for boys aged 13–15 and 7–18 years, girls aged 7–15 and 7–18 years improved, and there were some exceptions that speed of boys aged 7–12 and 16–18 years, girls aged 16–18 years had no significant changes (all *p* > 0.05) (Tables 1, 2). In terms of the rate of change, the rate of improvement in the 3^rd^ stage was higher for girls aged 7–18 years than in the 1^st^ stage, while the opposite was observed for boys. The improvement in the 3^rd^ stage for both boys and girls was mainly concentrated in the age categories of 13–15 years, and girls aged 7–12 years also improved to some extent (Fig. 7).

### Explosive power

**Figure 3:**
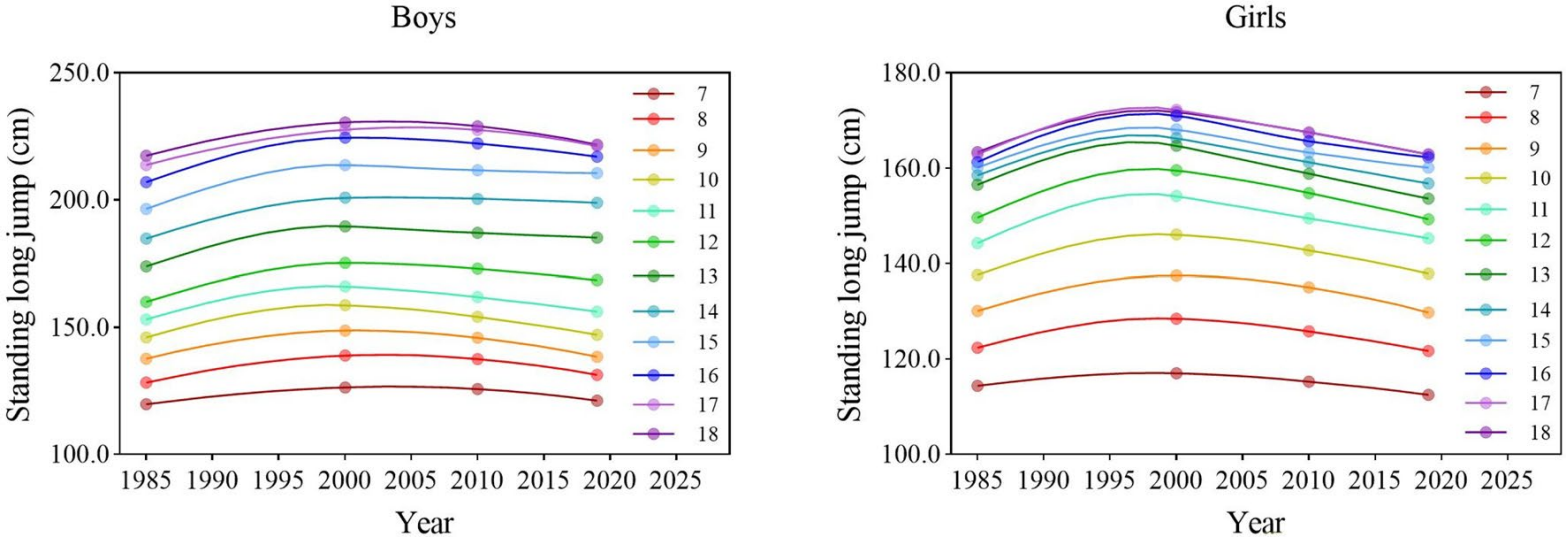
Secular trends (means) in standing long jump tests for Chinese boys and girls aged 7–18 in rural from 1985 to 2019. Dots and solid lines are the means and Akima splines, respectively. Upward sloping lines represent better performance over time and downward sloping lines represent poorer performance.

During the entire period, the explosive power of boys and girls in all age groups improved first then worsened (Fig. 3). There were significant differences in the results for SLJ of boys and girls between the four survey years [boys: *F* = 1694.614 (7–12 years), 1846.750 (13–15 years), 1890.707 (16–18 years), 1710.962 (7–18 years); girls: 860.430 (7–12 years), 691.314 (13–15 years), 822.869 (16–18 years), 1575.503 (7–18 years), all *p* < 0.001] (Tables 1, 2). In 2019, compared with 1985, the results for SLJ of boys in all age categories improved (all *p* < 0.05), while girls in general worsened (13–15 and 7–18 years, all *p* < 0.05) but the results for SLJ girls aged 7–12 and 16–18 years had no significant changes (all *p* > 0.05). From each period, during the period 1985–2000, the results for SLJ improved in all age categories and overall for both sexes (all *p* < 0.05); during the period 2000–2010, the results for SLJ for boys and girls in all age categories worsened (all *p* < 0.05) and continued to decline during the period 2010–2019 (Tables 1, 2). In terms of the rate of change, the rate of decline in the 3rd stage was higher for boys and girls aged 7–18 years than in the 2nd stage, especially in boys, and the rate of decline increased to more than 3 times in the 2nd stage. However, the rate of decline in the 3rd stage was significantly faster for boys aged 7–12 and 16–18 years than for boys aged 13–15 years, and the range of decline was very large compared to the 2nd stage. The rate of decline in the 3rd stage for boys aged 13–15 years increased only slightly compared to the 2nd stage. There was little difference in the rate of decline for girls in the three age categories in the 3rd stage (Fig. 7).

### Flexibility

**Figure 4:**
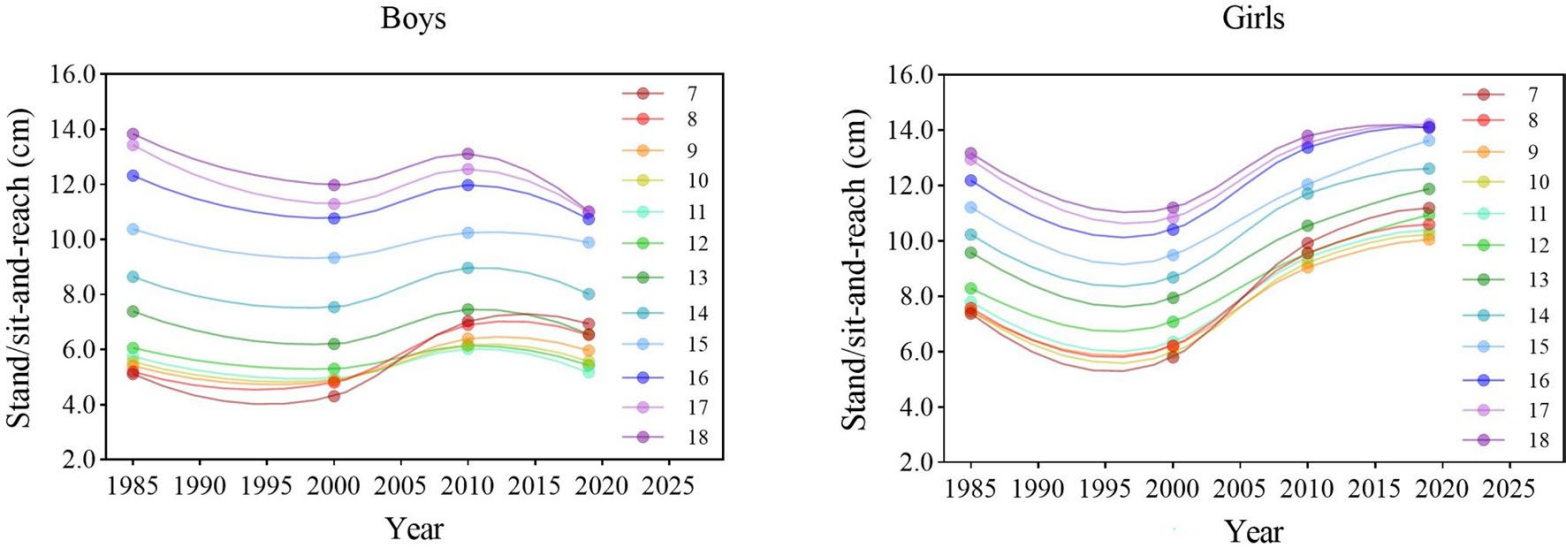
Secular trends (means) in stand/sit-and-reach tests for Chinese boys and girls aged 7–18 in rural from 1985 to 2019. Dots and solid lines are the means and Akima splines, respectively. Upward sloping lines represent better performance over time and downward sloping lines represent poorer performance.

During the entire period, the flexibility of boys in all age groups worsened first, then improved, and finally worsened. The flexibility of girls in all age groups worsened during the period 1985–2000 and then constantly improved after 2000. There were some differences among the different age groups (Fig. 4). There were significant differences in the results of SR of boys and girls between the four survey years [boys: *F* = 474.044 (7–12 years), 116.352 (13–15 years), 434.280 (16–18 years), 536.586 (7–18 years); girls: 3771.308 (7–12 years), 1027.921 (13–15 years), 717.771 (16–18 years), 4468.133 (7–18 years), all *p* < 0.001] (Tables 1, 2). In 2019, compared with 1985, the results for SR of boys aged 13–18 and 7–18 years worsened (all *p* < 0.05) but boys aged 7–12 years improved (*p* < 0.05). The results for SR of girls in all age categories improved (all *p* < 0.05). From each period, during the period 1985–2000, the results for SR worsened but improved during the period 2000–2010 in all age categories and overall for both sexes (all *p* < 0.05); during the period 2010–2019, the results for SR of boys in all age categories worsened but the opposite for girls (all *p* < 0.05) (Tables 1, 2). In terms of the rate of change, the rates of decline in the 3rd stage were higher for boys in all age categories and overall than in the 1st stage, especially in boys aged 16–18 years. Although the results for SR of girls in all age categories and overall continued to increase during the period 2000–2019, the rates of increase in the 3rd stage were not as fast as those in the 2nd stage (Fig. 7).

### Muscular strength

**Figure 5:**
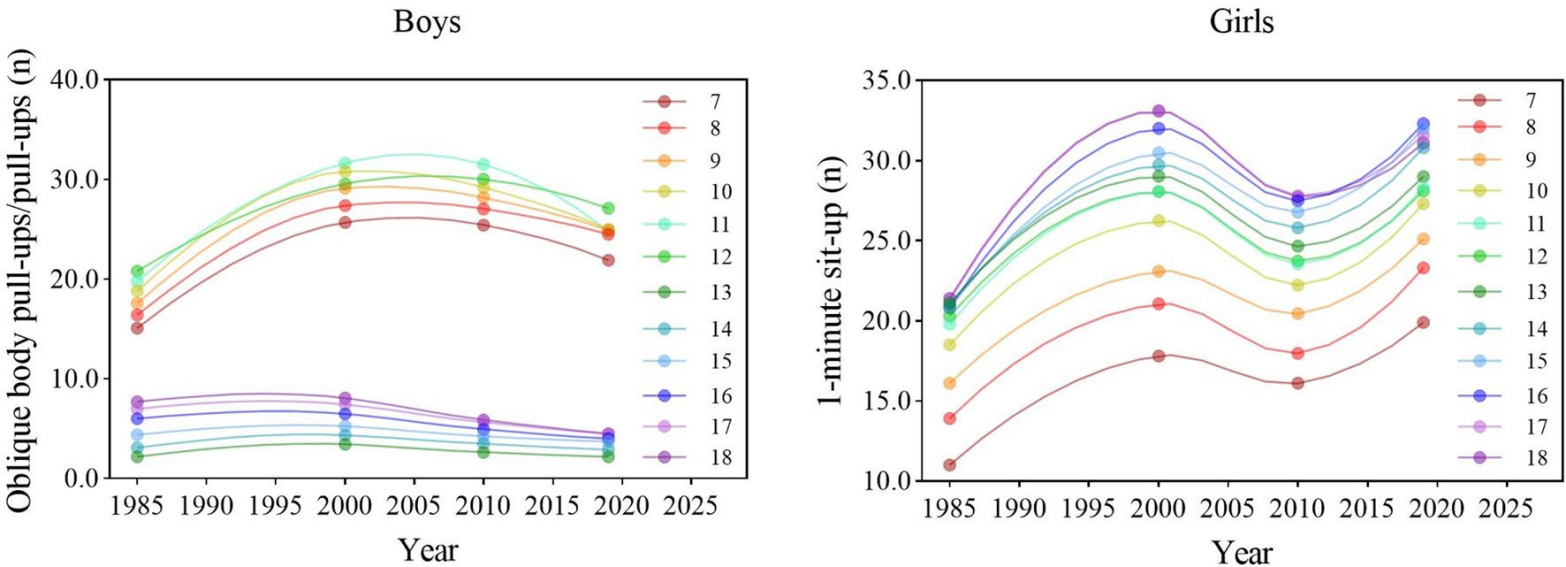
Secular trends (means) in muscular strength tests for Chinese boys (7–12-years-old: oblique body pull-ups; 13–18-years-olds: pull-ups) and girls (7–18-years-old: 1-min sit-ups) in rural from 1985 to 2019. Dots and solid lines are the means and Akima splines, respectively. Upwards sloping lines represent better performance over time and downwards sloping lines represent poorer performance.

During the entire period, the muscular strength of girls in all age groups improved first, then worsened, and finally improved. The muscular strength of boys in all age groups improved during the period 1985–2000 and then constantly worsened after 2000 (Fig. 5). There were significant differences in the results of OPU for boys aged 7–12 years, PU for boys aged 13–18 years and SU for girls between the four survey years [boys: *F* = 4028.403 (7–12 years), 313.363 (13–15 years), 1338.465 (16–18 years); girls: 4973.544 (7–12 years), 3467.113 (13–15 years), 5190.252 (16–18 years), 4468.133 (7–18 years), all *p* < 0.001] (Tables 1, 2). In 2019, compared with 1985, the results for OPU of boys aged 7–12 years improved but the results for PU of boys aged 13–18 years worsened (all *p* < 0.05). The results for SU of girls in all age categories and overall improved (all *p* < 0.05). From each period, during the period 1985–2000, the results for muscular strength tests for boys and girls improved but worsened during the period 2000–2010 in all age categories (all *p* < 0.05); during the period 2010–2019, the results for tests of boys in all age categories continued to worsen but the opposite for girls (all *p* < 0.05) (Tables 1, 2). In terms of the rate of change, the rate of increase in the 3rd stage was lower for girls in general than in the 1st stage but there was an exception in that of girls aged 7–12 years was faster. Although the results for tests of boys in all age categories continued to decline in the 3rd stage, the decline accelerated for boys aged 7–12 years while the decline slowed for boys aged 13–18 years compared to the 2nd stage (Fig. 7).

### Cardiorespiratory fitness

**Figure 6:**
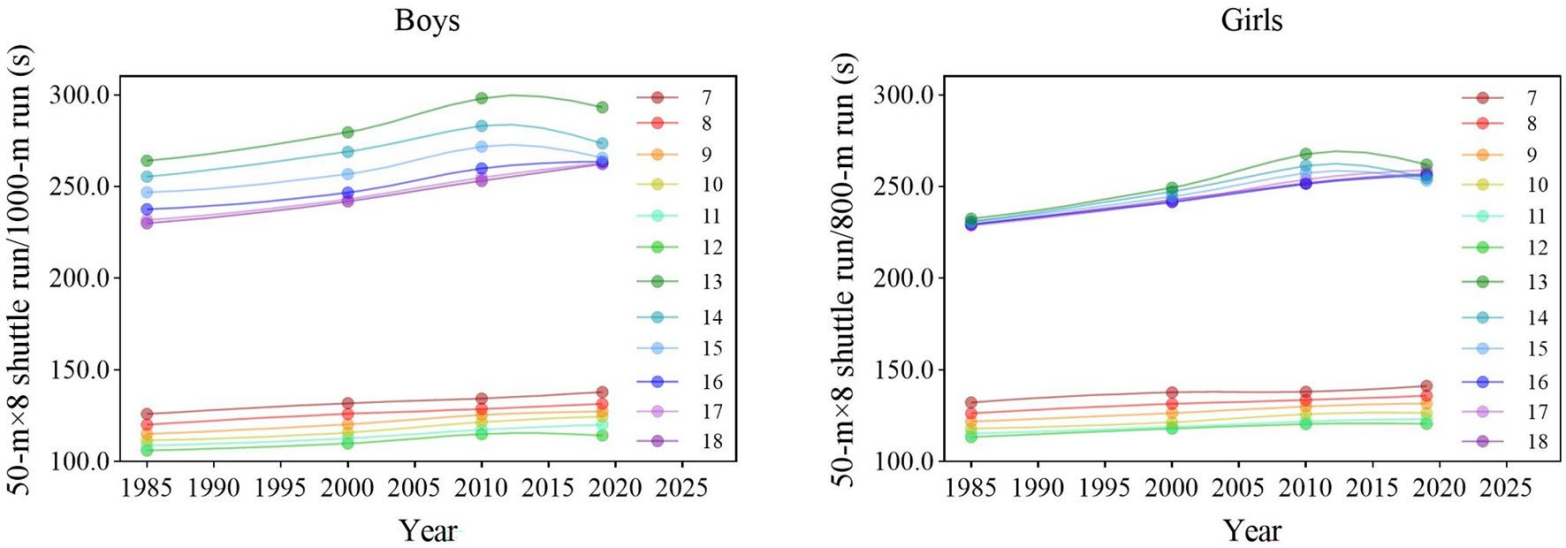
Secular trends (means) in cardiorespiratory fitness tests for Chinese boys (7–12-years-olds: 50-m × 8 shuttle run; 13–18-years-olds: 1000-m run) and girls (7–12-years-olds: 50-m × 8 shuttle run; 13–18-years-olds: 800-m run) in rural from 1985 to 2019. Dots and solid lines are the means and Akima splines, respectively. Upward sloping lines represent poorer performance over time and downward sloping lines represent better performance.

**Figure 7:**
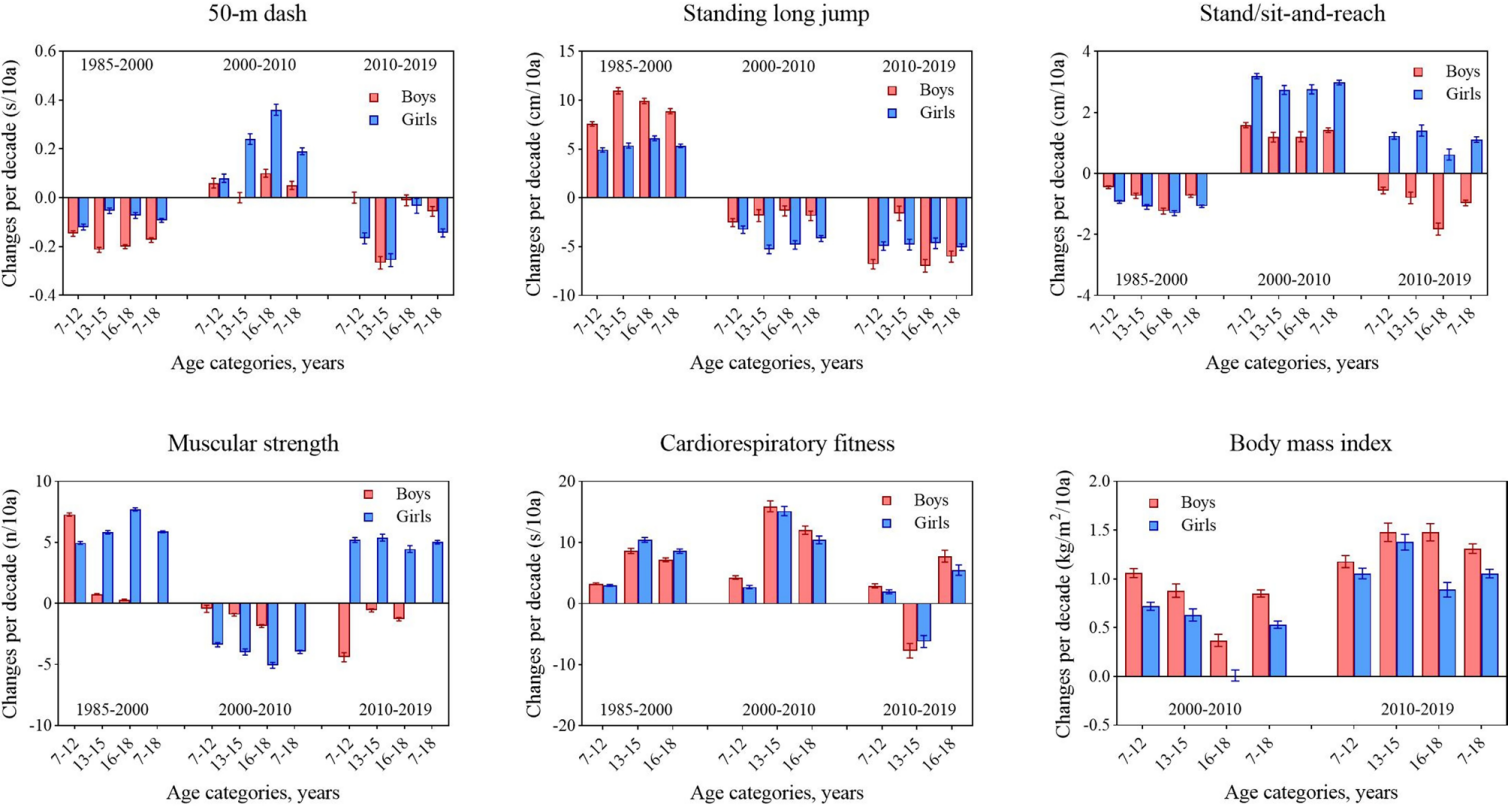
Change in the mean (95% confidence interval) differences in the physical fitness test results of Chinese rural children and adolescents in different age categories and genders from 1985 to 2019 per decade. Muscular strength is assessed by oblique body pull-ups for boys aged 7–12, pull-ups for boys aged 13–18, and 1-min sit-ups for girls aged 7–18 years; cardiorespiratory fitness is assessed by 50 m × 8 shuttle run for boys and girls aged 7–12, a 1000-m running for boys aged 13–18, and a 800-m running for girls aged 13–18. From 1985 to 2000, the flexibility test was stand-and-reach. Since 2005, sit-and-reach has been used to measure flexibility.

During the entire period, the cardiorespiratory fitness of boys and girls in most age groups worsened (the means of tests increased) (Fig. 6). There were significant differences in the results of 50SR for boys and girls aged 7–12 years, 1000R for boys aged 13–18 years and 800R for girls between the four survey years [boys: *F* = 41,119.917 (7–12 years), 2320.016 (13–15 years), 3894.106 (16–18 years); girls: 2495.489 (7–12 years), 3704.561 (13–15 years), 3903.492 (16–18 years), 4468.133 (7–18 years), all *p* < 0.001] (Tables 1, 2). Boys and girls aged 7–12 and 16–18 years showed linear upward trends in the results of tests over 34 years (all *p* < 0.05), which represented the decline in cardiorespiratory fitness. In 2019, compared with 1985, the cardiorespiratory fitness of boys and girls in all age categories worsened (the means of 50SR, 1000R and 800R increased) (all *p* < 0.05). From each period, during the period 1985–2000 and 2000–2010, the cardiorespiratory fitness for boys and girls in all age categories worsened (all *p* < 0.05); during the period 2010–2019, the cardiorespiratory fitness of boys and girls 7–12 and 16–18 years continued to worsen but improved for boys and girls 13–15 years (all *p* < 0.05) (Tables 1, 2). In terms of the rate of change, the rate of decline in the 3rd stage was lower for boys and girls aged 7–12 and 16–18 years than in the 2nd stage. The rate of decline in the 2nd stage was highest for boys and girls in general (Fig. 7).

## Discussion

The results showed that during the period 1985–2000, the physical fitness of Chinese rural children and adolescents improved in most aspects except for flexibility, and most of them reached the largest increases; during the period 2000–2010, except for flexibility, all other fitness decreased significantly; during the period 2010–2019, the speed of boys rebounded and flexibility, explosive power and muscular strength continued to decline. The largest rates of decline in fitness were reached in general except for muscular strength in boys aged 13–18 years. Speed, flexibility and muscular strength of girls rebounded and explosive power continued to decline. The cardiorespiratory fitness of boys and girls had significant downward trends in general during the entire period but improved significantly for boys and girls in junior middle school, and the decline slowed down in primary school and junior high school. The largest increases in BMI in boys and girls occurred in the period between 2010 and 2019. In general, some components of fitness of Chinese children and adolescents in rural has shown positive trends in recent years, especially for girls.

The speed for both sexes improved after 2010, which was similar to other Chinese studies^2,11,12,35^ and a Japanese study (2013–2019)^36^, which both demonstrated positive trends in recent years. Some studies found that Chinese urban children and adolescents also show positive trends in speed after 2010 as well, but positive trends were more pronounced in rural areas^11,12^. Speed in children and adolescents aged 8–15 years in Mozambique, Africa, declined continuously from 1992 to 2012^37^. The trends in Slovenia are almost consistent with our study^15^, with speed quality first decreasing and then rebounding. However, studies from some developed countries^6,38–42^ showed different results that speed remained stable in children after 2000, having previously improved or worsened or stabilized. There are some exceptions, such as the decline in speed of Portuguese girls from 2003/2008 to 2008/2013^38^ and in the Netherlands from 2006 to 2015/2017^43^. Our study also found that during the period 2010–2019, the speed of adolescents aged 16–18 years had no significant changes, and some studies also provided some evidence^35,44^. A systematic review by Fühner et al.^45^ showed that speed in children and adolescents had been increasing since 2002 and declined to a minimum in the 1980s, while rural Chinese boys bottomed out in 1985 and girls in 2010. Other systematic reviews, which were not quantified, showed inconsistent trends in speed across countries^1,44^. Moreover, we found significant differences in speed items between countries, with straight-line dashes (e.g., 30/50/60-m dash) being related to the ability to move quickly; speed-agility (e.g., 10 × 5 m shuttle run) also included the ability to change body position/orientation quickly and accurately in response to stimuli^46^. Three types of speed were selected in Italy, and the results of dashes remained almost constant over the last 30 years, but the shuttle run declined^40^. Therefore, we estimate that the differences in secular trends in speed may be related to the test items, although it is known that speed is highly genetically determined^1^. In short, the current worldwide change trends of speed vary across the world.

The explosive power of boys and girls began to improve in 1985 but worsened after 2000, and the rate of decline increased. The trend was similar to other Chinese studies^2,11,12,35^ in Chinese urban areas^11,12^. However, we found that the explosive power of rural boys improved from 2015 to 2018 in Shanghai, China^13^, and improved in Japan in the last decade^36^. Some developed countries, such as Slovenia (until 2014)^21^, Lithuania^39^, Italy^41^, Poland^47^, and Brazil^48^, reported negative trends at the beginning of the twenty-first century or throughout the observed period, with many of these studies showing no significant changes^15,39,41,47^ or improvement for girls in recent years^21^. The explosive power of Croatian and German boys had no significant changes and improved for girls slightly since the beginning of the twenty-first century^14,49^. Slovenia had stable or positive trends (especially for girls) from 2014 to 2019 after the previous downward trend^15^. Positive trends were observed among boys and girls in the United States in the twentieth century and in Greece in the early twenty-first century^50,51^. Tomkinson et al. converged on the standing long jump performance of 10,940,801 children and adolescents from 29 countries during the period 1960–2017 and found that the rate of improvement was steady from the 1960s to the 1980s, slowed in the 1990s, and then declined thereafter^20^, which is generally consistent with the results of this paper. In addition to the above results using the standing long jump as an explosive power test, we also found that the performance of continuous leaping (Finland)^52^, horizontal jump (Portugal)^38^, high jump (Netherlands)^43^, and vertical jump (rural Poland)^6^ worsened or remained stable or in recent years stabilized^52^ and, in general, did not show positive trends. Several reviews also pointed to the negative trends of explosive power in most countries^1,44^, but we found that explosive power remained stable in children and adolescents (especially girls) in some countries around the 2110s. Meanwhile, we found downward trends for girls and boys, and the decline for boys accelerated, suggesting the need to focus on explosive power in boys in China and globally.

We observed that flexibility worsened during the period 1985–2000 and then improved during the period 2000–2010 for both sexes, but the trends were different for boys (worsened) and girls (improved) during the period 2010–2019. Overall, the flexibility worsened in boys and improved in girls. The trends were similar to nationwide research and Chinese urban areas^11^, but there were different trends in some regions of China (first stable then worsened or improved)^13,35^. The flexibility of boys worsened and improved in girls in Hong Kong from 1998 to 2015^53^ which was similar to our findings A 30-year negative trend was observed in Africa^37^, but a positive trend was observed in Japan in the previous decade^36^. For some developed countries, such as Croatia^14^, Portugal^38^, Lithuania^39^, rural Germany^42^, Dutch^43^, Poland (Krakow)^47^, and Brazil^48^, a large number of studies have shown that flexibility has worsened. Other studies (e.g., rural Poland^6^, Slovenia^15^, Italy^41^, Germany^49^, Canada^54^, and Brazil^55^) found negative or steady trends in boys and steady or positive trends in girls in recent years. Meanwhile, an upward trend was found in Greece for both sexes^50^. In conclusion, most of the studies found that flexibility worsened, and there were positive trends for girls in recent years which was consistent with this study. In addition, we also observed a significant decline in higher-age boys, the exact reasons for which need to be explored in depth.

The trends of muscular strength showed differences among boys and girls with boys having mostly negative trends and girls having mostly positive trends during the period 1985–2019. Muscular strength in boys worsened and in girls improved in recent years. The trends were similar to nationwide research and Chinese urban areas^2,11,12^. However, Xinjiang, China, found positive trends in recent years only in boys aged 13–18 and worsened in both boys and girls in Hong Kong (sit-ups for both sexes)^35,53^, which differs from this study and suggests differences between different regions of China. The performance of sit-ups improved in Japan but declined after 2019^36^. In some developed countries, increasing or stable sit-up performance was observed in Croatia^14^, Slovenia^15^, Greece^50^ Portugal^38^ and Germany^42,49,56^, and negative trends were found only in Brazil^48,55^ and the United Kingdom^57^. On the other hand, for the bent-arm hangs test, we found that most studies showed downward trends, such as in Slovenia^15^, Dutch^43^, Lithuania^39^, and the UK^57^. We also found a decline in backward overhead medicine ball throws in Poland (Krakow)^47^. Kaster et al.^19^ estimated secular trends of sit-up performance for 9,939,289 children and adolescents aged 9–17 years from 31 countries/regions from 1964 to 2017 and found that most countries showed positive trends. Although there was a negative international trend after 2010, the lack of data for a large number of developing countries made the interpretation of the results incomplete^19^. These different secular trends might be related to the specifics of the different tests used for muscle strength, such as bent-arm hang/flexed-arm hang, pull-up, sit-up, handgrip, etc., which emphasize arm and shoulder belt strength, abdomen strength, etc. Meanwhile, the selection of tests varies between countries and is not always consistent between boys and girls.

Cardiorespiratory fitness significantly worsened in Chinese rural children and adolescents. The negative trends were similar to nationwide researchs and Chinese urban areas^2,11,35,53^, but improvements were also observed for children aged 7–12 years from 2005 to 2014^11,12^. Cardiorespiratory fitness improved in Japan but worsened after 2019^36^. Previous studies have shown negative trends in cardiorespiratory fitness in children and adolescents in many countries around the world^2,11,13,14,37,39,48,54,55^ and some studies have found that cardiorespiratory fitness has been stable (boys or girls or both sexes) in recent years^35,41,42,49,56^. However, there are also studies reporting positive trends, such as Greece^51^, rural Poland (girls)^6^, and Slovenia^15,58^. Fühner et al.^45^ and Tomkinson et al.^19^ identified stabilization and possible improvement after 2010 or 2000.

During the period 2000–2019, the BMI of boys and girls in all age groups increased and the increase accelerated, especially in those aged 16–18 years. It has been shown that both higher and lower BMI can have a detrimental effect on physical fitness^22,59^. Overweight and obesity individuals tend to perform less physical activity, have longer screen time, consume more calories^60^ and have a substantial decrease in physical activity. The study from CNSSCH showed that the prevalence and increased rate of overweight and obesity among rural Chinese boys were higher than those among girls^24^, which may be one of the reasons for the more obvious improvement in girls' physical fitness in recent years. However, a slow or stagnant or negative increase in the prevalence of overweight and obesity was observed in recent years^61,62^, which partly explains the improvements in some physical fitness in children and adolescents during the period 2010–2019. In addition, some studies have noted that nutritional status is an important factor affecting physical fitness regardless of the levels of physical activity^63^, and Dong et al.^64^ also found that children and adolescents with high level of physical activity and high socioeconomic status were associated with better physical fitness, and children and adolescents with obesity and longer TV viewing time were associated with worse physical fitness. Most of these factors were independently and significantly associated with physical fitness. Physical fitness is influenced by several factors and there are also some interactions among these factors, but nutritional status might be the key factor of physical fitness in children and adolescents. In addition, children and adolescents in lower age groups were less resistant to food temptations than those in higher age groups^60^, and their physical inactivity was more likely to lead to overweight or obesity^65^.

It was worth noting that the secular trends in some fitness (cardiorespiratory fitness, speed, muscular strength) were found to be more “positive” for both sexes during junior middle school than for other educational phases. Other studies did not demonstrate such distinctly different trends across age categories as this paper (possibly due to the age range limitations of the participants or the lack of focus on age differences), and some worldwide studies found more positive trends in sit-ups (with a smaller rate of decline) in recent years in children than in adolescents^19^, while cardiorespiratory fitness showed almost no difference^18^. Since participants of our study were divided based on the Chinese educational stage and observed significantly different trends for this age category, we believe that it might be related to some Chinese-specific factors. One study found that the increased rate of overweight and obesity among Chinese adolescents aged 14–17 years was smaller than that of children aged 7–13 years in recent years^61^, while a study from CNSSCH (Henan, China) showed a rapid increase in the prevalence of overweight and obesity in students aged 10–12 and 16–18 years from 2010 to 2019^66^. From our study, we found that the increase in BMI was faster in adolescents at higher ages from 2010 to 2019, but these findings cannot fully explain the positive trend of adolescents aged 13–15 years in recent years. Therefore, we estimate that another occurrence of this phenomenon is related to the junior high school entrance examination for physical education. Piloting from the twenty-first century, 31 administrative districts in China included the exam of physical education in the total score (physical education and culture scores) of the junior high school examination, signifying the full implementation that the exam of physical education was included among the junior high school entrance examination; initially, the exam of physical education accounted for 5% of the total score of the junior high school entrance examination, and then, in response to the decline in the physical fitness of students nationwide and the need for sports power strategy, the score of the physical education examination was constantly increased (other cultural scores remained almost unchanged) and even doubled in some districts^67^. In addition, the test programs have become more diverse, but the 1000-m running for boys and 800-m running for girls belong to the mandatory test programs, which may explain the significant improvements in cardiorespiratory fitness in adolescents aged 13–15 years. Although we are unclear how this initiative has impacted them (e.g., voluntary or pressured participation in physical activity), it is clear from the CNSSCH that from 2005 to 2014, the rates of the good and excellent health status of physical fitness rose significantly for 13–15 years than for 16–18 years^68,69^. On the other hand, they proves some evidence. In contrast, the National College Entrance Examination (NEMT or Gaokao) and junior middle school entrance examination do not include physical education subjects or do not count toward the total score, and students are not highly motivated to exercise. Meanwhile, students at junior high school are under more academic pressure and spend more time being sedentary,^70^, which may worsen their health status.

In the early twenty-first century, the nation and society have paid great attention to the decrease in physical fitness of children and adolescents, and a series of initiatives have been taken. For example, the Healthy Physical Education Curriculum Model of China^71^, developed by Professor Ji, was introduced to improving students' physical fitness by helping them enjoy physical education and engage in at least one sport as a hobby. The curriculum must focus on the three key elements of "sports loading, physical fitness training and motor skills". Students should have approximately 10 min of physical readiness training in each session, and the exercise intensity should be at least 75% in each session, with an average heart rate of 140–160 beats per minute per session. Currently, the model has been promoted nationwide and a large number of Chinese physical education teachers have been trained to use the model^35,72^. The government implemented the Opinions of the Central Committee of the Communist Party of China on Strengthening Youth Sports to Enhance the Physical Fitness of Young People in 2007^45^ while emphasizing the importance of school physical education and ensuring that students exercise for one hour every day at school. For the first time, Children and adolescents’ sports health promotion has been elevated to the level of national strategy. In the years that followed, the government issued more than 88 policies including the promotion of sports, reductions in academic burden, and promotion of physical fitness^2,73^. To improve the health of poor rural students, the state launched the "Nutrition improvement program for rural compulsory education students" in 2011, with the financial department providing nutritional meal subsidies for rural compulsory education students (approximately 7–15 years) in poor areas of central and western areas. In the beginning, each student was provided with a subsidy of 3 yuan per study day, which was increased to 4 yuan in 2014 and 5 yuan in 2021, and the subsidy amounted to 34.8 billion yuan in 2021^74,75^. Through this, the average height and weight of students increased, and the gap between urban and rural areas narrowed; micronutrient deficiencies such as anemia decreased; the intake of foods rich in high-quality protein and micronutrients such as fish, poultry, meat, eggs and milk increased, and rural students’ nutrition levels improved^74,75^. A study from CNSSCH found an increase in the percentage of children and adolescents meeting one hour of in-school physical activity^76^. These findings may be related to improvements in physical fitness in recent years. In addition, the Central Committee of the Communist Party of China and the State Council issued the "Outline of the Healthy China 2030 Plan" to improve the physical fitness of the whole population as one of the strategic goals in 2016^77^. In 2019, the Health China Action Promotion Committee issued the "Health China Action (2019–2030)"^78^, which clearly states that by 2022 and 2030, the proportion of students meeting the national physical fitness standards (National student physical health standard, revised in 2014) will reach at least 50% and 60%, respectively. The Physical Education Law of the People's Republic of China, which is amended by the Standing Committee of the 13th National People's Congress on June 24, 2022, stipulates that physical education subjects will be included in NEMT from January 2023^79^. We predict that in the future the physical fitness of Chinese children and adolescents will show more positive trends.

Our study spans more than three decades and a long time interval, providing not only a report of secular trends in physical fitness but also an exploration of changes in different year phases. Since there are differences between urban and rural areas, such as economic and political, separate analyses of the physical fitness of rural children and adolescents are beneficial for the development of future rural promotion strategies. In contrast to including only a few age groups, this study includes 12 age groups for each sex from childhood to adulthood and is divided again according to educational phases, which facilitates the characteristics of physical fitness in boys and girls at different stages of growth and development, and our study did find different secular trends in cardiorespiratory fitness. This study has several limitations. First, the muscular strength and cardiorespiratory fitness tests differ across age categories for boys and girls, which does not facilitate comparisons between them. Then, the assessment of rural area being done only in 1985 and urban areas are also possibly included in later study waves. This may increase the differences in economics, policies, etc. between the rural areas selected, and secular trends may not be applicable to all rural areas. Finally, it has been demonstrated that physical activity, nutritional status, and dietary habits can have an effect on physical fitness, and this study did not include these variables or conduct a correlation study.

## Conclusion

Our results are nearly consistent with previous studies in China, and it complements the most recent data and evidence from rural areas. Our study showed that from 1985 to 2019, although the physical fitness of children and adolescents in rural China previously experienced negative trends, some components of physical fitness have begun to improve in recent years. At the same time, we also found that certain physical fitness have shown negative trends in recent years, with varying trends for gender and age subgroups. This implies that despite favorable trends over the past decade, there are inequalities in the physical fitness development of Chinese children and adolescents, which may also contribute to future health inequalities, pointing to the need for China to focus on physical fitness and health equity among children and adolescents in the future. Preferential policies for rural areas, promoting physical activity, reducing academic pressures, reducing sedentary time and preventing obesity could all be effective countermeasures.

## Data Availability

The datasets used and/or analyzed during the current study are available from the corresponding author on reasonable request.

## References

[1] Masanovic B (2020). Trends in physical fitness among school-aged children and adolescents: A systematic review. Front Pediatr..

[2] Dong Y (2019). Trends in physical fitness, growth, and nutritional status of Chinese children and adolescents: A retrospective analysis of 1·5 million students from six successive national surveys between 1985 and 2014. Lancet. Child. Adolese..

[3] Zhao MM, Zhou ZT, Sun YX, Li J (2022). Correlation between physical fitness and blood pressure in school aged children. Chin. J. Prev. Contr. Chron. Dis..

[4] Ortega FB, Ruiz JR, Labayen I, Lavie CJ, Blair SN (2018). The Fat but Fit paradox: What we know and don’t know about it. Br. J. Sports. Med..

[5] Henriksson P (2022). Body composition, physical fitness and cardiovascular risk factors in 9-year-old children. Sci. Rep..

[6] Bartkowiak S (2021). Physical fitness of rural polish school youth: Trends between 1986 and 2016. J. Phys. Act. Health..

[7] Zhang YH, Sun JZ, Li N (2016). Analysis of height and weight growth changes of children and adolescents in China from 1943 to 2014. Chin. J. Sch. Health..

[8] Ji CY, Hu PJ, He ZH (2007). Secular growth trends in the Chinese urban youth and its implications on public health. J. Peking. Univ. Health Sci..

[9] Liu ZM, Yang SR, Fang JQ, Li X (2017). Long-term trend of growth and development for primary and middle school students in China from 1985 to 2014. Mod. Prev. Med..

[10] Wang S, Dong YH, Wang ZH, Zhou ZY, Ma J (2017). Trends in overweight and obesity among Chinese children of 7–18 years old during 1985–2014. Chin. J. Prev. Med..

[11] Wu J, Yuan SM (2019). Dynamic analysis of physical function and fitness of Chinese students from 1985 to 2014. J. Beijing. Sport. Univ..

[12] Ao D, Wu F, Yun CF, Zheng XY (2019). Trends in physical fitness among 12-year-old children in urban and rural areas during the social transformation period in China. J. Adolesc. Health..

[13] Yan YJ, Yan YL (2021). Changes of health and physical fitness of children and adolescents in Shanghai, 2015–2018. Mod. Prev. Med..

[14] Kasović M, Štefan L, Petrić V (2021). Secular trends in health-related physical fitness among 11–14-year-old Croatian children and adolescents from 1999 to 2014. Sci. Rep..

[15] Radulović A, Jurak G, Leskošek B, Starc G, Blagus R (2022). Secular trends in physical fitness of Slovenian boys and girls aged 7 to 15 years from 1989 to 2019: A population-based study. Sci. Rep..

[16] Huotari P, Gråstén A, Huhtiniemi M, Jaakkola T (2022). Secular trends in 20 m shuttle run test performance of 14- to 15-year-old adolescents from 1995 to 2020. Scand. J. Med. Sci. Sports..

[17] Dooley FL (2020). A systematic analysis of temporal trends in the handgrip strength of 2,216,320 children and adolescents between 1967 and 2017. Sports. Med..

[18] Tomkinson GR, Lang JJ, Tremblay MS (2019). Temporal trends in the cardiorespiratory fitness of children and adolescents representing 19 high-income and upper middle-income countries between 1981 and 2014. Br. J. Sports. Med..

[19] Kaster T (2020). Temporal trends in the sit-ups performance of 9,939,289 children and adolescents between 1964 and 2017. J. Sports. Sci..

[20] Tomkinson GR (2021). Temporal trends in the standing broad jump performance of 10,940,801 children and adolescents between 1960 and 2017. Sports. Med..

[21] Đurić S (2021). Secular trends in muscular fitness from 1983 to 2014 among Slovenian children and adolescents. Scand. J. Med. Sci. Sports..

[22] Chen G, Chen J, Liu J, Hu Y, Liu Y (2022). Relationship between body mass index and physical fitness of children and adolescents in Xinjiang, China: A cross-sectional study. BMC Public Health.

[23] Hsu CY (2021). Can anthropometry and body composition explain physical fitness levels in school-aged children?. Children (Basel)..

[24] Song Y (2019). National trends in stunting, thinness and overweight among Chinese school-aged children, 1985–2014. Int. J. Obes (Lond).

[25] Central People's Government of the People's Republic of China. Opinions on implementing the strategy of rural revitalization (accessed 18 September 2022); http://www.gov.cn/zhengce/2018-02/04/content_5263807.htm (2018–02–04).

[26] Central People's Government of the People's Republic of China. Decided to launch and implement the nutrition improvement plan for rural compulsory education students (accessed 18 September 2022); http://www.gov.cn/ldhd/2011-10/26/content_1979016.htm. (2011–10–26).

[27] Cheng WL (2021). Height, weight and prevalence of overweight and obesity among 10–15 years old children in China, 2010–2016. Chin. J. Public. Health..

[28] Lu YH (2022). The association of different sedentary patterns and health-related physical fitness in Pre-schoolers. Front. Pediatr..

[29] Lang JJ (2022). Top 10 international priorities for physical fitness research and surveillance among children and adolescents: A twin-panel delphi study. Sports. Med..

[30] CNSSCH Association. *Report on the 1985th National Survey on Students' Constitution and Health*. (People's Educational Publication, 1987).

[31] CNSSCH Association. *Report on the 2000th National Survey on Students' Constitution and Health*. (Higher Educational Press, 2000).

[32] CNSSCH Association. *Report on the 2010th National Survey on Students' Constitution and Health*. (Higher Educational Press, 2012).

[33] CNSSCH Association. *Report on the 2019th National Survey on Students' Constitution and Health*. (Higher Educational Press, 2022).

[34] Morgan SL, Dutt AK, Costa FJ, Aggarwal S, Noble AG (1994). The impact of the growth of township enterprises on Rural-Urban transformation in China. The Asian City: Processes of Development, Characteristics and Planning. The GeoJournal Library, 30.

[35] Bi C, Zhang F, Gu Y, Song Y, Cai X (2020). Secular trend in the physical fitness of Xinjiang children and Adolescents between 1985 and 2014. Int. J. Environ. Res. Public. Health..

[36] Kidokoro T, Tomkinson GR, Lang JJ, Suzuki K (2022). Physical fitness before and during the COVID-19 pandemic: Results of annual national physical fitness surveillance among 16,647,699 Japanese children and adolescents between 2013 and 2021. J. Sport. Health. Sci..

[37] Dos Santos FK (2012). Secular trends in physical fitness of Mozambican school-aged children and adolescents. Am. J. Hum. Biol..

[38] Costa AM, Costa MJ, Reis AA, Ferreira S, Martins J, Pereira A (2017). Secular trends in anthropometrics and physical fitness of young Portuguese school-aged children. Acta. Med. Port..

[39] Venckunas T, Emeljanovas A, Mieziene B, Volbekiene V (2017). Secular trends in physical fitness and body size in Lithuanian children and adolescents between 1992 and 2012. J. Epidemiol. Community. Health..

[40] Vandoni M (2022). The temporal association between body characteristics and speed performance over twenty-five years in Italian adolescents. Children (Basel)..

[41] Lovecchio N, Giuriato M, Carnevale Pellino V, Valarani F, Codella R, Vandoni M (2020). Italian physical fitness decline: A true fact or a mindset? A 10-year observational perspective study. Int. J. Environ. Res. Public. Health..

[42] Eberhardt T, Bös K, Niessner C (2022). Changes in physical fitness during the COVID-19 pandemic in German children. Int. J. Environ. Res. Public. Health..

[43] Anselma M, Collard DCM, van Berkum A, Twisk JWR, Chinapaw MJM, Altenburg TM (2020). Trends in Neuromotor fitness in 10-to-12-year-old Dutch children: A comparison between 2006 and 2015/2017. Front. Public. Health..

[44] Eberhardt T (2020). Secular trends in physical fitness of children and adolescents: A review of large-scale epidemiological studies published after 2006. Int. J. Environ. Res. Public. Health..

[45] Fühner T, Kliegl R, Arntz F, Kriemler S, Granacher U (2021). An update on secular trends in physical fitness of children and adolescents from 1972 to 2015: A systematic review. Sport. Med..

[46] Giuriato M, Codella R, Lovecchio N, Carnevale Pellino V, Vandoni M, Nevill AM (2021). Speed agility trends in children according to growth. Ann. Hum. Biol..

[47] Kryst Ł, Żegleń M, Artymiak P, Kowal M, Woronkowicz A (2022). Analysis of secular trends in physical fitness of children and adolescents (8–18 years) from Kraków (Poland) between 2010 and 2020. Am. J. Hum. Biol..

[48] Gaya AR (2020). Temporal trends in physical fitness and obesity among Brazilian children and adolescents between 2008 and 2014. J. Hum. Sport. Exerc..

[49] Hanssen-Doose A (2021). Population-based trends in physical fitness of children and adolescents in Germany, 2003–2017. Eur. J. Sport. Sci..

[50] Smpokos EA, Linardakis M, Papadaki A, Lionis C, Kafatos A (2012). Secular trends in fitness, moderate-to-vigorous physical activity, and TV-viewing among first grade school children of Crete, Greece between 1992/93 and 2006/07. J. Sci. Med. Sport..

[51] Pinoniemi BK, Tomkinson GR, Walch TJ, Roemmich JN, Fitzgerald JS (2021). temporal trends in the standing broad jump performance of United States children and Adolescents. Res. Q. Exerc. Sport..

[52] Jaakkola T, Gråsten A, Huhtiniemi M, Huotari P (2021). Changes in the continuous leaping performance of Finnish adolescents between 1979 and 2020. J. Sports. Sci..

[53] Poon ET, Tomkinson G, Huang WY, Wong SHS (2022). Temporal trends in the physical fitness of Hong Kong adolescents between 1998 and 2015. Int. J. Sports. Med..

[54] Colley RC (2019). Trends in physical fitness among Canadian children and youth. Health. Rep..

[55] Blasquez Shigaki G, Batista MB, Paludo AC, Vignadeli LFZ, Serassuelo Junior H, Ronque ERV (2019). Secular trend of physical fitness indicators related to health in children. J. Hum. Growth. Dev..

[56] Spengler S, Rabel M, Kuritz AM, Mess F (2017). Trends in motor performance of first graders: A comparison of cohorts from 2006 to 2015. Front. Pediatr..

[57] Sandercock GRH, Cohen DD (2019). Temporal trends in muscular fitness of English 10-year-olds 1998–2014: An allometric approach. J. Sci. Med. Sport..

[58] Morrison SA, Sember V, Leskošek B, Kovač M, Jurak G, Starc G (2021). Assessment of secular trends and health risk in pediatric cardiorespiratory fitness from the Republic of Slovenia. Front. Physiol..

[59] Qin G, Qin Y, Liu B (2022). Association between BMI and health-related physical fitness: A cross-sectional study in Chinese high school students. Front. Public. Health..

[60] Zhu SQ, Zhang YJ (2022). Analysis of behavioral risk factors for overweight and obesity among children and adolescents (7–17 years old) in China. Chin. J. Prev. Contr. Chron. Dis..

[61] Guo Y, Yin X, Wu H, Chai X, Yang X (2019). Trends in overweight and obesity among Children and adolescents in China from 1991 to 2015: A meta-analysis. Int. J. Environ. Res. Public. Health..

[62] Hu X (2022). Trends of overweight and obesity among children and adolescents aged 7–17 in 16 provinces of China from 2000 to 2018. J. Hyg. Res..

[63] Malicevic S (2022). Is the physical fitness of schoolchildren dependent on their physical activity levels and nutritional status? The experience from Serbia. Nutr. Hosp..

[64] Dong X (2021). Physical activity, screen-based sedentary behavior and physical fitness in Chinese adolescents: A cross-sectional study. Front. Pediatr..

[65] Ren SS (2022). Correlation between physical activity and nutritional status among Chinese Children and adolescents. Chin. J. Sch. Health.

[66] Zhang Y (2022). Trends of overweight and obesity prevalence in school-aged children among Henan Province from 2000 to 2019. Front. Public. Health..

[67] Bai YL, Nie RX, Li XD (2022). Historical changes, evolutionary characteristics and future directions of the reform of physical education for junior high school entrance examination. Chin. Exam..

[68] Song Y (2022). Trends of prevalence of excellent health status and physical fitness among Chinese Han students aged 13 to 18 years from 1985 to 2014. Beijing Da Xue Xue Bao Yi Xue Ban.

[69] Zhang JS (2020). Analysis on the trend of prevalence of excellent and good physical fitness and health status among Chinese Han students aged 13 to 18 years and related influencing factors from 1985 to 2014. Zhonghua Yu Fang Yi Xue Za Zhi.

[70] Wang D (2017). Improving school physical education to increase physical activity and promote healthy growth of Chinese school-aged children-time for action. J. Sport. Health. Sci..

[71] Dong CX, Lv HM (2020). Theoretical basis and practical basis for establishing key points of the Healthy Physical Education Curriculum Model of China. Chin. Sport. Sci..

[72] Ji L (2019). A Re-study on the theoretical and practical problems of healthy physical education curriculum model of China. J. Beijing. Sport. Univ..

[73] Dong Y (2021). Individual-, family-, and school-level ecological correlates with physical fitness among Chinese school-aged children and adolescents: A national cross-sectional survey in 2014. Front. Nutr..

[74] Ma L (2022). National childhood obesity-related intervention systems and intervention programs in China in 1949 to 2020: A narrative review. Obesity (Silver Spring).

[75] Zhang Q (2022). A decade of review and prospects for improving the nutrition and health of Chinese primary and secondary school students. J. Hyg. Res..

[76] Yan X (2020). Comparison of status of physical activity time at school and influencing factors in students in China, 2010 and 2014. Zhonghua Liu Xing Bing Xue Za Zhi.

[77] Central Committee of the Communist Party of China and the State Council. Outline of the Healthy China 2030 Plan (accessed 18 September 2022); http://www.gov.cn/xinwen/2016‑10/25/content_5124174.htm (2016‑10‑25).

[78] Health China Action Promotion Committee. Health China Action (2019–2030) (accessed 18 September 2022); http://www.gov.cn/xinwen/2019-07/15/content_5409694.htm (2019‑07‑15).

[79] General Administration of Sport. The Physical Education Law of the People's Republic of China is amended (accessed 18 September 2022); https://www.sport.gov.cn/n31/n20067006/c24405447/content.html (2022‑06‑25).

